# Highly Potent GalNAc-Conjugated Tiny LNA Anti-miRNA-122 Antisense Oligonucleotides

**DOI:** 10.3390/pharmaceutics13060817

**Published:** 2021-05-31

**Authors:** Tsuyoshi Yamamoto, Yahiro Mukai, Fumito Wada, Chisato Terada, Yukina Kayaba, Kaho Oh, Asako Yamayoshi, Satoshi Obika, Mariko Harada–Shiba

**Affiliations:** 1 Graduate School of Biomedical Sciences, Nagasaki University, Nagasaki 852-8521, Japan; bb55620011@ms.nagasaki-u.ac.jp (C.T.); bb30218011@ms.nagasaki-u.ac.jp (Y.K.); bb30118006@ms.nagasaki-u.ac.jp (K.O.); asakoy@nagasaki-u.ac.jp (A.Y.); 2 Graduate School of Pharmaceutical Sciences, Osaka University, Osaka 565-0871, Japan; mkyh0804@gmail.com (Y.M.); fumitism@gmail.com (F.W.); obika@phs.osaka-u.ac.jp (S.O.); 3 Department of Molecular Innovation in Lipidology, National Cerebral and Cardiovascular Center Research Institute, Suita, Osaka 564-8565, Japan; mshiba@ncvc.go.jp

**Keywords:** tiny LNA, miR-122, GalNAc, Ligand-targeted drug delivery system, antisense oligonucleotide

## Abstract

The development of clinically relevant anti-microRNA antisense oligonucleotides (anti-miRNA ASOs) remains a major challenge. One promising configuration of anti-miRNA ASOs called “tiny LNA (tiny Locked Nucleic Acid)” is an unusually small (~8-mer), highly chemically modified anti-miRNA ASO with high activity and specificity. Within this platform, we achieved a great enhancement of the in vivo activity of miRNA-122-targeting tiny LNA by developing a series of *N*-acetylgalactosamine (GalNAc)-conjugated tiny LNAs. Specifically, the median effective dose (ED50) of the most potent construct, **tL-5G3**, was estimated to be ~12 nmol/kg, which is ~300–500 times more potent than the original unconjugated tiny LNA. Through in vivo/ex vivo imaging studies, we have confirmed that the major advantage of GalNAc over tiny LNAs can be ascribed to the improvement of their originally poor pharmacokinetics. We also showed that the GalNAc ligand should be introduced into its 5′ terminus rather than its 3′ end via a biolabile phosphodiester bond. This result suggests that tiny LNA can unexpectedly be recognized by endogenous nucleases and is required to be digested to liberate the parent tiny LNA at an appropriate time in the body. We believe that our strategy will pave the way for the clinical application of miRNA-targeting small ASO therapy.

## 1. Introduction

MicroRNAs (miRNAs) are ubiquitously expressed, ~22-nucleotide single-stranded non-coding RNAs that play a fundamental role in the post-transcriptional regulation of gene expression in cells [1]. The mature miRNA, a guide strand RNA, is taken up by a member of the Argonaut (AGO) protein family, while the other passenger strand undergoes degradation. AGO uses the MID and PIWI domains to pre-organize a seed region of miRNA (7–8 bases from the second 5′ end of miRNA) to facilitate binding to the target mRNAs. In general, the 3′-untranslated region of mRNA contains miRNA target sites that are highly complementary to the seed region. Accumulating evidence suggests that miRNAs are involved in the development and progression of a variety of human diseases, such as viral infections, cancer, cardiovascular diseases, and neurodegenerative diseases. miRNAs have been recognized as attractive drug targets since their discovery [2]. In particular, attempts to inhibit miRNAs by nucleic acid oligomers such as miRNA-sponge, miRNA-masking, and anti-miRNA antisense oligonucleotides (ASO), including antimir and antagomir strategies, have so far been successfully demonstrated, in which various chemical modifications such as 2′-*O*-methyl (2′-OMe) RNA, 2′-*O*-methoxyethyl (2′-MOE) RNA, and 2′,4′-BNA/LNA have been utilized to functionally fortify this class of drugs [3].

miR-122, a liver-specific miRNA associated with cholesterol and lipid metabolism as well as hepatitis C virus (HCV) replication [4,5,6,7,8,9], has been anticipated as a promising drug target suitable for ASO therapy; however, none of the potential clinical candidates have been successful to date [4,10,11,12,13,14]. Significant improvements in the safety and selectivity of this class of agents are still required for their subsequent clinical applications. In this context, LNAs (also known as 2′,4′-BNAs) have been shown to be useful artificial nucleic acid building blocks that unprecedentedly enhance the binding affinity and sequence selectivity of ASOs to target RNA, making them a promising drug discovery platform. Of note, a specific configuration called tiny LNA, first introduced by Kauppinen et al. in 2011, is a seed-complementary, fully LNA-modified, unusually small (~8-mer) anti-miRNA ASOs with high activity and specificity [15]. A single tiny LNA can simultaneously target a specific seed match family of genes; thus far, several different miRNAs, such as miR-21, miR-122, and miR-33a/b, have been validated as targets that showed significant therapeutic effects in animals [15,16]. However, the systemically administered tiny LNAs can be widely distributed in tissues and organs throughout the body; therefore, avoiding off-target distribution of the tiny LNAs by precision drug delivery systems would be of critical importance with respect to both maximizing their potency and reducing their side effects.

GalNAc is a well-recognized carbohydrate ligand of the liver-specific asialoglycoprotein receptor (ASGPR), which is highly expressed in hepatocytes and exhibits a fast turnover rate, making the ligand–receptor system suitable for liver targeting [17]. In this context, we and others have recently reported an approach for the hepatocyte delivery of ASO or siRNA drugs by developing a unique monomer-type *N*-acetylgalactosamine (GalNAc) ligand specialized for oligonucleotide-based drugs [18,19,20]. Among the congeners of conjugatable GalNAc ligands reported so far, the monovalent GalNAc phosphoramidite unit, including ours, has some advantages such as a shorter synthetic scheme, solid-phase synthesis applicability, and increased freedom in configuration design. In previous studies, we demonstrated the usefulness and flexibility of this type of GalNAc unit on gapmer-type ASOs and optimized the structure of the building block to maximize the effect of the ligand [18,21,22]. Specifically, we have realized that a biolabile phosphodiester linkage is more favorable than a biostable phosphorothioate bond as a ligand-connecting linker, indicating that after being taken up by the hepatocytes, the conjugate should be digested quickly to liberate its parent ASO, although some endonucleases are thought to be responsible for this cleavage. In this respect, it is also quite intriguing to know how the linker structure of the GalNAc unit affects the potency of the tiny LNA, which is less likely to be a substrate of the hypothetical endonuclease(s).

Here, we first demonstrate the in vivo activity of GalNAc-conjugated anti-miR122 tiny LNAs and how the ligand structure and configuration affect the in vivo potency of their conjugates.

## 2. Materials and Methods

### 2.1. Design and Synthesis of ASOs

The hydroxy-L-prolinol-based GalNAc (GalNAc*_hp_*) phosphoramidite unit was synthesized according to a previous report [23]. Unless otherwise specified, all the 2′,4′-BNA/LNA-based ASOs shown in Table 1 were synthesized by Gene Design Inc. (Osaka, Japan). The sequence of the tiny LNA was designed to be complementary to the seed region of miRNA-122, which is common between humans and mice, according to a previous report [15].

For in-house synthesis of oligonucleotides, all phosphoramidite monomers were dried over P_2_O_5_ in a desiccator under vacuum overnight, and MeCN was dehydrated using MS3A prior to use. The LNA-T-phosphoramidite and LNA-A (Bz)-phosphoramidite monomers were purchased from FUJIFILM Wako Pure Chemical Corporation (Osaka, Japan). The LNA-C (Bz)-phosphoramidite was purchased from Merck (Darmstadt, Germany). DNA synthesis reagents used in this study were purchased from Glen Research (Sterling, VA, USA). The GalNAc*_hp_* phosphoramidite monomer was dissolved in dry MeCN to a final concentration of 0.1 M, and other phosphoramidite monomers were dissolved in dry MeCN to a final concentration of 0.067 M. The synthesis of oligonucleotides (**tinyCtr-5G3**) was performed at a 0.2-µmol scale using an automated DNA synthesizer (NST M-2-TRS, Nihon Techno Service Co., Ltd., Ibaraki, Japan) with 0.25 M 5-benzylthio-1*H*-tetrazole as an activator. 3′-Amino linker oligonucleotides were synthesized at a 1.0-µmol scale on 3′-PT-Amino-Modifier C6 controlled pore glass (CPG) purchased from Glen Research (Sterling, VA, USA) by using the automated DNA synthesizer with 0.25 M 5-benzylthio-1*H*-tetrazole in MeCN as an activator. The GalNAc*_hp_* phosphoramidite was manually incorporated into oligonucleotides with 0.25 M 5-ethylthio-1*H*-tetrazole as an activator using standard phosphoramidite chemistry. The DMTr-protected ASOs were treated with 28% NH_3_ aq. at 55 °C for 12–14 h, in which step oligomers were cleaved from the CPG and all the protecting groups except the terminal DMTr group were removed. The crude ASOs were purified by Glen-Pak™ DNA purification cartridge from Glen Research (Sterling, VA, USA). The 5′-DMTr group was removed using 4% (*v/v*) aqueous trifluoroacetic acid during this purification step. The resulting ASOs were further purified by reverse-phase HPLC (CTO-20AC/SPD-M10AVP/DGU-20A3/FCV-10ALVP for the equipment from Shimadzu, Kyoto, Japan) using a COSMOSIL 5C18-MS-II Packed column (10 mmI.D. × 250 mm, Nakarai tesque, Inc., Kyoto, Japan) under a following condition: A = 100 mM HFIP 8 mM TEA, B = MeOH, 5–30% of B in 30 min, flow 1.5 mL/min. The purity of the materials was analyzed using analytical reverse-phase HPLC using a COSMOSIL 5C18-MS-II Packed column (4.6 mmI.D. × 50 mm, Nakarai tesque, Inc., Kyoto, Japan) under a following condition: A = 100 mM HFIP 8 mM TEA, B = MeOH, 5–40% of B in 20 min, flow 1.0 mL/min (Figure S1). The identification was carried out using MALDI-TOF-MS. The MALDI-TOF mass data [M+Na] were as follows; **tinyCtr-5G3**, found 4199.87, sodium adduct (calcd 4200.47).

### 2.2. Methods for 3′ Labeling of Oligonucleotides with Alexa Fluor 647 Dye

Alexa Fluor™ 647 labeled ASOs (**tinyLNA-f, tL-5G3-f**) were synthesized via a conjugation reaction where each 3′-amino linker ASO (**tinyLNA**, **tL-5G3**) in 1x D-PBS (–) (FUJIFILM Wako Pure Chemical Co., Osaka, Japan) (pH = 7.1–7.7) was treated with 5 equivalents of Alexa Fluor™ 647 NHS esters (Fisher Scientific, Slangerup, Denmark). The reaction mixture was agitated for 22 h at a final concentration of 0.2 M. The labeled ASOs were purified using NAP-5 Columns (Cytiva, Marlborough, MA, USA). The ASOs were further purified by reverse-phase HPLC (COSMOSIL 5C18-MS-II Packed, 10 mmI.D. × 250 mm, A = 100 mM HFIP 8 mM TEA, B = MeOH, 5–40% of B in 30 min, flow 3.0 mL/min). Buffer exchange into saline was performed using NAP-5 Columns. Analytical HPLC was performed for the sample (COSMOSIL 5C18-MS-II Packed, 4.6mmI.D. × 50 mm, A = 100 mM HFIP 8 mM TEA, B = MeOH, 5–40% of B in 20 min, flow 1.0 mL/min, Figures S2 and S3) and the identification was carried out using MALDI-TOF-MS. **tinyLNA-f**: Found 3846.78, Calcd 3843.47, **tL-5G3-f**: Found 5289.41, Calcd 5289.97.

### 2.3. Animal Study

All animal procedures were performed with the consent of the Animal Care Ethics Committees of the National Cerebral and Cardiovascular Center Research Institute (Osaka, Japan) (Approval numbers: 20076 and 21003, Date of Approval: 12 January 2021 and 1 April 2021, respectively) and Nagasaki University (Nagasaki, Japan) (Approval numbers: 1911011572-1~3 and 1911061573-1~3, Date of Approval: 2 November 2019 and 6 November 2019, respectively). All mice were 6-week-old male C57Bl/6J strains purchased from SLC Japan (Tokyo, Japan). All studies were initiated when the mice were 8 weeks old. The mice were kept on a 12-h light/dark cycle with free access to food and water. The mice were fed normal chow (CE-2, CLEA Japan, Tokyo, Japan) for one week prior to the experiments. ASOs solution or control saline was administered to the mice subcutaneously at a dose range of 10–3 nmol/kg. At 1 week post-injection, the mice were anesthetized with isoflurane (Escain^®^, Pfizer Japan, Tokyo, Japan) and then sacrificed. Peripheral blood was collected in BD Microtainer^®^ tubes (BD, Franklin Lakes, NJ, USA). Liver tissues were harvested and stored in RNAlater^®^ (Thermo Fisher Scientific, Slangerup, Denmark) or snap-frozen in liquid nitrogen.

### 2.4. Serum Chemistry

Peripheral blood collected from sacrificed mice in BD Microtainer^®^ tubes were centrifuged at 5000× *g* for 20 min at 4 °C to obtain the serum. Serum levels of total cholesterol, total high-density lipoprotein (HDL), aspartate aminotransferase (AST), alanine aminotransferase (ALT), and creatinine (CRE) were measured using a DRI-CHEM 7000 chemistry analyzer (Fujifilm, Tokyo, Japan).

### 2.5. RNA Isolation from Mice Liver

For quantitative RT-PCR, total RNA was isolated using the Quick Gene RNA Tissue S-II kit (Kurabo, Osaka, Japan). For the evaluation of miR-122 inhibition based on the mobility shift assay or SplintR^®^ Ligase (New England BioLabs, Ipswich, MA, USA), total RNA was isolated using TRIzol Reagent (Thermo Fisher Scientific) with one modification as described below. During the precipitation step, a mixture of 250 µL of isopropanol and 250 µL of salt solution (0.8 M sodium citrate, 1.2 M) was added to the aqueous phase instead of 500 µL of isopropanol.

### 2.6. Quantitative Real-Time PCR

cDNA was synthesized from 1–3 µg of total RNA using the High Capacity cDNA Reverse Transcription Kit (Applied Biosystems, Foster City, CA, USA). Quantitative PCR was performed using the Fast SYBR Green system (Applied Biosystems, Foster City, CA, USA). The expression levels of each target gene were normalized to those of *GAPDH* mRNA. The primers used for real-time PCR were as follows: Aldoa forward, 5-acattgctgaagcccaacat-3; Aldoa Reverse, 5-acaggaaagtgaccccagtg-3; Bckdk Forward, 5-tgatgctctattccggtcgc-3; Bckdk reverse, 5-ttgatgcggtgagcaatcct-3; Aldob Forward, 5- ccgcttgcaggaacaaacaa-3; and Aldob reverse, 5-acgccacttcccaaagtcaa-3′.

### 2.7. Evaluation of miR-122 Inhibition Using SplintR^®^ Ligase

Total RNA was diluted to a concentration of 5 ng/µL and mixed with one pair of DNA probe spanned miR-122. For annealing, the mixture was incubated at 95 °C for 5 min and then at 50 °C for 40 min. Then, for ligation, the mixture was added with SplintR^®^ Ligase (New England BioLabs, Ipswich, MA, USA) and 1,4-dithiothreitol was added to the mixture, followed by incubation at 37 °C for 14 h. The ligated DNA probes were quantified using TaqMan Fast Master Mix (Applied Biosystems, Foster City, CA, USA) to indirectly calculate the abundance of miR-122 [24].

### 2.8. Evaluation of Inhibition via Mobility Shift Assay

Total RNA (25 µg) and FAM-conjugated **Anti-miR122-probe** (25 or 42 fmol) were mixed in a buffer (50% (*v*/*v*) glycerol, 0.01% (*v*/*v*) bromophenol blue, 1 TBE). Each mixture was incubated at 60 °C for 30 min and then chilled on ice for 3 min. The samples were separated by electrophoresis using a 20% TBE Gel (Novex, MA, USA). Bands were visualized using an Image Quant LAS4000 (GE Healthcare, Chicago, IL, USA) system with a Y515-Di filter and analyzed using ImageJ 1.53a (https://imagej.nih.gov/ij/, accessed on 20 April 2021).

### 2.9. In Vivo/Ex Vivo Studies

Balb/cSlc-nu/nu 6-week-old male mice were purchased from SLC Japan (Tokyo, Japan). Mice were kept in standard rodent cages in temperature-controlled rooms under a 12-h light/dark cycle with free access to water and food in a specific-pathogen-free animal facility at Nagasaki University. Mice were fed an autofluorescence-reduced diet (D10001, Research Diets Inc., New Brunswick, NJ, USA) for more than one week before the dosing study was commenced. Alexa Fluor^TM^ 647-labeled **tinyLNA-f** and **tL-5G3-f** were dissolved in saline (Otsuka Normal Saline, Otsuka Pharmaceutical Co. Ltd., Tokyo, Japan) for injection. Mice were intravenously injected with a single dose of each oligonucleotide (300 pmol in 100 μL saline) via the tail vein, and biodistribution was visualized using an IVIS Lumina II imaging system (Caliper Life Science, Hopkinton, MA, USA; excitation filter, 640 nm; emission filter, Cy5.5., exposure time = 5 s) at several different time points up to 90 min. Mice were anesthetized with isoflurane (Escain^®^, Pfizer Japan, Tokyo, Japan) while taking snapshots. Mice were then euthanized and subjected to systemic perfusion with saline (10 mL). Excised tissues from the six mice were washed by saline, displayed on a cell culture dish, and snapshots were taken for fluorescent images as well as ROI (regions of interest) measurement analysis on IVIS Lumina II (Caliper Life Science, Hopkinton, MA, USA).

### 2.10. Statistical Analysis

Data are presented as mean ± SD. Statistical analyses were performed using either Holm’s t-test or Dunnett’s multiple comparisons test to assess the statistical significance of the results obtained against the saline-treated control arms unless otherwise specified. * *p* < 0.05, ** *p* < 0.01. “ns” stands for not statistically significant.

## 3. Results and Discussions

### 3.1. Effect of GalNAc Conjugation on In Vivo Activity of Tiny LNA

**Figure 1:**
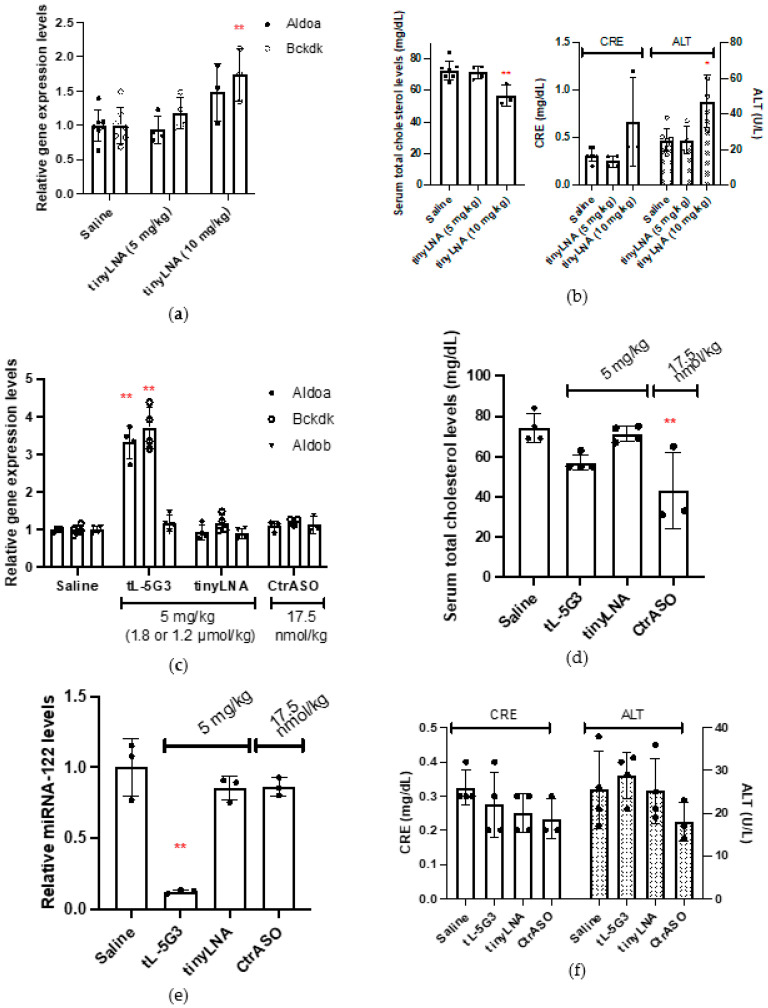
In vivo evaluation of effect of GalNAc conjugation on ASOs’ activity. (**a**) The expression levels of miRNA-122-targeted mRNAs in the livers of mice (6-week-old male C57Bl6/J, *n* = 3–6) after 7 days of injection at a dosage of 5 mg/kg or 10 mg/kg of **tinyLNA**. (**b**) Their corresponding serum cholesterol levels and kidney and liver transaminase levels. (**c**) The expression levels of miRNA-122-targeted mRNAs in the livers of mice (6-week-old male C57Bl6/J, *n* = 3–4) after 7 days of injection at a dosage of 5 mg/kg for **tinyLNA** and **tL-5G3** or 17.5 nmol/kg for **CtrASO**. (**d**–**f**) Their corresponding serum cholesterol levels, miRNA-122 expression levels, and kidney and liver transaminase levels, respectively. ** *p* < 0.01 and * *p* < 0.05 (vs. saline).

First, to estimate the minimum amount of the antimiR-122 tiny LNA required for the onset of the pharmacological effect, we injected mice subcutaneously with two doses of 5 mg/kg or 10 mg/kg of **tinyLNA** (Table 1) as described by Kauppinen et al., as a potent anti-mir-122 tiny LNA [15], and the mice were sacrificed on day 7. Quantitative RT-PCR analysis of liver RNA revealed that at a dose of 5 mg/kg, the expression levels of two known miR-122 target mRNAs, Aldorase A (Aldoa) and Branched-chain α-ketoacid dehydrogenase complex (Bckdk), did not change upon treatment, while the expression level of both mRNAs were found to increase upon administration of a 10-mg/kg dose (Figure 1a). Despite showing no statistical difference, the expression of Bckdk at 5 mg/kg and Aldoa at 10 mg/kg seemed to respond to the intervention. Blood cholesterol concentrations were also evaluated, as it is well known that miRNA-122 interferes with cholesterol metabolism [4], and it showed a significant reduction only after treatment at 10 mg/kg (Figure 1b). On the other hand, a slight elevation of the levels of liver and kidney enzymes (creatinine or CRE, and aspartate aminotransferase or ALT) in the blood were found after administration of a 10-mg/kg dose, indicating the likelihood of liver and kidney injury (Figure 1b). We did not observe any significant increase in the levels of the two miRNA-122-targeted mRNAs, Aldoa and Bckdk, in the **tinyLNA**-treated group, probably since the dose administered was below the lowest dosage compared to a previous report, where at least three consecutive doses of 5 mg/kg of **tinyLNA** were injected. Overall, we confirmed that the pharmacological activity of tiny LNA started at approximately 5–10 mg/kg, thus we selected 5 mg/kg as the starting dose for the following study.

Subsequently, to confirm whether the GalNAc conjugation strategy works for the tiny LNA, which is a fully chemically modified, extraordinarily short oligonucleotide structurally dissimilar to both native DNA and RNA, we prepared two additional oligonucleotides for comparison: **CtrASO** and **tL-5G3**, as listed in Table 1. **CtrASO**, used here as a GalNAc-bearing control no sequence matched with miRNA-122, is an apolipoprotein B (apoB) mRNA-targeting gapmer-type ASO, previously described as a potent cholesterol-lowering agent [18]. Comparing the ability to reduce cholesterol with this well-described ASO is informative when considering the development of the antimir-122 tiny LNA as a cholesterol-lowering agent. **tL-5G3** is an miR122-targeting tiny LNA carrying three GalNAc*_hp_* units on its 5′ terminus. The GalNAc*_hp_* units were loaded onto the tiny LNA through a conventional phosphoramidite solid-phase DNA synthesis method, in which a phosphodiester bond was adopted (rather than a phosphorothioate bond) as the linker chemistry of an inter-GalNAc*_hp_* and ASO-GalNAc*_hp_* based on our previous notion of gapmer-type ASOs [18,21]. Mice were subcutaneously injected with **CtrASO** at a single dose of 17.5 nmol/kg and with **tinyLNA** or **tL-5G3** at a single dose of 5 mg/kg (1.84 and 1.20 µmol/kg, respectively) and sacrificed 7 days after injection. We selected the dosage in the present study by referring to the original literature on tiny LNAs. We then examined the effect of these ASOs on gene expression and blood chemistry profile with those of the saline-treated control. Quantitative RT-PCR analysis of each RNA isolated from the livers of the mice revealed a three- to four-fold increase in the miRNA-122 target Aldoa, whereas Bckdk mRNAs were observed only in the GalNAc-conjugated **tL-5G3**-treated arm, while the expression of the non-target Aldorase B (Aldob) mRNA remained unchanged in this arm (Figure 1c) [25]. We did not observe any significant increase in the levels of the two miRNA-122-targeted mRNAs, Aldoa and Bckdk, in the **tinyLNA**-treated group at this dose, as mentioned earlier. Blood cholesterol analysis showed that **tL-5G3** reduced blood cholesterol levels by 23% (*p* = 0.0623), while **tinyLNA** did not alter the serum total cholesterol concentration (Figure 1d), which was consistent with the mRNA profile obtained. It should be noted that **CtrASO** largely reduced serum cholesterol, while the expression levels of these two miRNA-122-targeted mRNAs and the control Aldob mRNA remained unchanged (Figure 1c,d). Even under conditions where **tL-5G3** has a maximum effect, its cholesterol-lowering ability was still lower than that of the apoB-targeting **CtrASO**.

To further confirm the effect of GalNAc-conjugated **tL-5G3** on the upregulation of mRNA expression and cholesterol lowering, we indirectly quantified the amount of miRNA-122 using a SplintR^®^ ligase-based qPCR method [24]. Briefly, the method involves a two-step procedure: (1) ligation of two synthetic DNA fragments to make a single-stranded DNA probe, which was splinted with miRNA-122 using SplintR^®^ ligase, a Chlorella virus DNA ligase; and (2) the ligated DNA probe was then PCR-amplified and used to quantify miRNA-122 expression using a conventional real-time PCR method. One of the two DNA fragments is designed to bind to the 5′ end of miRNA-122, where its seed region is located; therefore, it cannot bind to the miRNA when ASO occupies the site; therefore, we can only detect the ASO-unbound fraction of miRNA-122. As shown in Figure 1e, only GalNAc-conjugated **tL-5G3** exhibited complete sequestration (88%) of miRNA-122, while other ASOs, including **tinyLNA**, did not affect the miRNA-122 expression level. We confirmed that the series of changes associated with **tL-5G3** shown in Figure 1c,d are in line with the efficient sequestration of miRNA-122 by **tL-5G3**. No hepatotoxicity or kidney injury associated with the injection of **tL-5G3** was found in the serum liver transaminase and creatinine analysis (Figure 1f), while at a dose of 10 mg/kg, **tinyLNA** showed a trend of liver and kidney injury (Figure 1b), whose toxic potential was also partially observed in previous studies [15]. The improved knockdown of miRNA-122 in the liver can be ascribed to an efficient shift of its biodistribution towards the liver, which can also lead to a reduction in its accumulation in the kidneys, resulting in a reduction of renal toxicity [22,26]. Another known benefit of GalNAc conjugation is its ability to mitigate off-target-associated hepatotoxicity [27]. The cellular uptake of GalNAc conjugates is mediated by ASGPR, the receptor of which is expressed specifically in liver parenchymal cells. Therefore, we conclude that GalNAc conjugation successfully elicited the in vivo potential of tiny LNAs, presumably by changing the pharmacokinetics of tiny LNAs, specifically localizing the drug to the liver parenchymal cells.

### 3.2. Evaluation of Dose Responsiveness of In Vivo Activity of GalNAc-Conjugated Tiny LNAs

**Figure 2:**
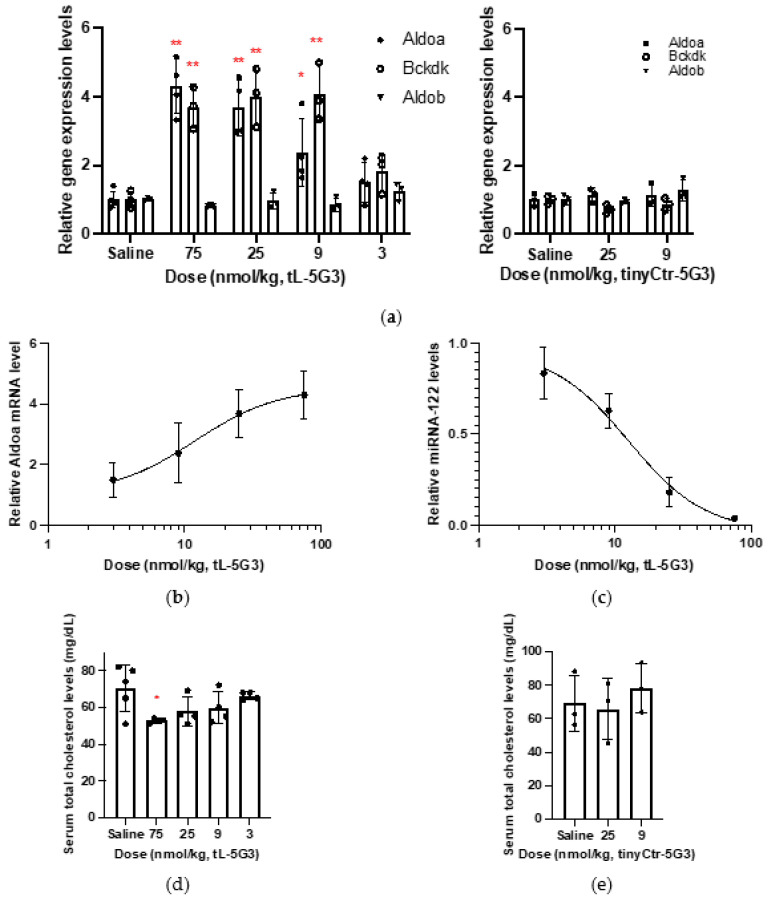
In vivo dose-responsiveness study of **tL-5G3**. (**a**,**b**) The hepatic expression levels of miRNA-122-targeted or non-targeted mRNAs in mice (6-week-old male C57Bl6/J, *n* = 3–4) after 7 days of injection at a dosage of 75–3 nmol/kg of **tL-5G3** and 25–9 nmol/kg of **tinyCtr-5G3**. (**c**,**d**) Their corresponding miRNA-122 expression levels and serum cholesterol levels, respectively. (**e**) Serum total cholesterol levels of the **tinyCtr-5G3**-treated arms. ** *p* < 0.01 and * *p* < 0.05 (vs. saline).

It seemed that the dose applied to mice at 5 mg/kg (=1200 nmol/kg) in the last section was more than that required for **tL-5G3**. Subsequently, to assess the dose responsiveness, each group of mice received each of several different doses ranging from 600 to 3 nmol/kg of **tL-5G3**, after 7 days of which mRNA and serum cholesterol profiles were analyzed. The highest level of increase in Aldoa and Bckdk mRNA was observed at a dose of approximately 75, 25, or even 9 nmol/kg for **tL-5G3**, while no significant effect of a sequence-scrambled GalNAc-conjugated tiny LNA, **tinyCtr-5G3**, was found on the gene expression levels of Aldoa, Bckdk, and Aldob at both 9 and 25 nmol/kg (Figure 2a). The ED50 value estimated from the Aldoa levels of **tL-5G3** was ~12 nmol/kg (Figure 2b). In line with this, a clear dose-dependent reduction of miRNA-122 expression level was observed, from which the ED50 was estimated to be approximately 12 nmol/kg, almost identical to that obtained from the surrogate marker Aldoa mRNA (Figure 2c). As expected, serum cholesterol reduction also showed a dose-dependent effect (Figure 2d), while no significant cholesterol change was observed in the **tinyCtr-5G3**-treated groups (Figure 2e). Overall, a clear dose-responsiveness was observed for **tL-5G3**, and together with Kauppinen’s previous study [15], we estimated that 12 nmol/kg (ED50) **tL-5G3** would be equivalent to 10–15 mg/kg (3680–5520 nmol/kg) unconjugated **tinyLNA**, which falls within the range of a ~300–500-fold molar ratio. This large improvement in potency was more than we had anticipated, given the current knowledge regarding GalNAc-conjugated gapmer-type ASOs [28]. Eventually, we realized that the optimal dose range of **tL-5G3** was ~10 nmol/kg, which is almost the same as that of **CtrASO**. By comparing these two molecules, we were able to confirm that neither GalNAc units nor LNA units affected the miR-122 profile and mRNA profile as themselves, and these pharmacological effects can be mainly ascribed to the tiny LNA’s on-target effect (Figure 1c,e vs. Figure 2). Thus, this salient benefit of GalNAc conjugation on tiny LNAs could be ascribed to the poorer tissue uptake of parent **tinyLNA** (8-mer) compared to that of gapmer ASOs (generally 15–20-mer phosphorothioate ASO) [29]. The latter are larger and more hydrophobic, and are thought to accumulate more naturally in the liver without ligand conjugation, while shorter phosphorothioate oligonucleotides are generally less likely to bind to plasma proteins, which are supposed to accelerate their elimination from the circulation [29,30,31]. Further experimental support is required to uncover the underlying mechanisms that provide this important benefit.

### 3.3. Visualization of miRNA-122 in Liver

**Figure 3:**
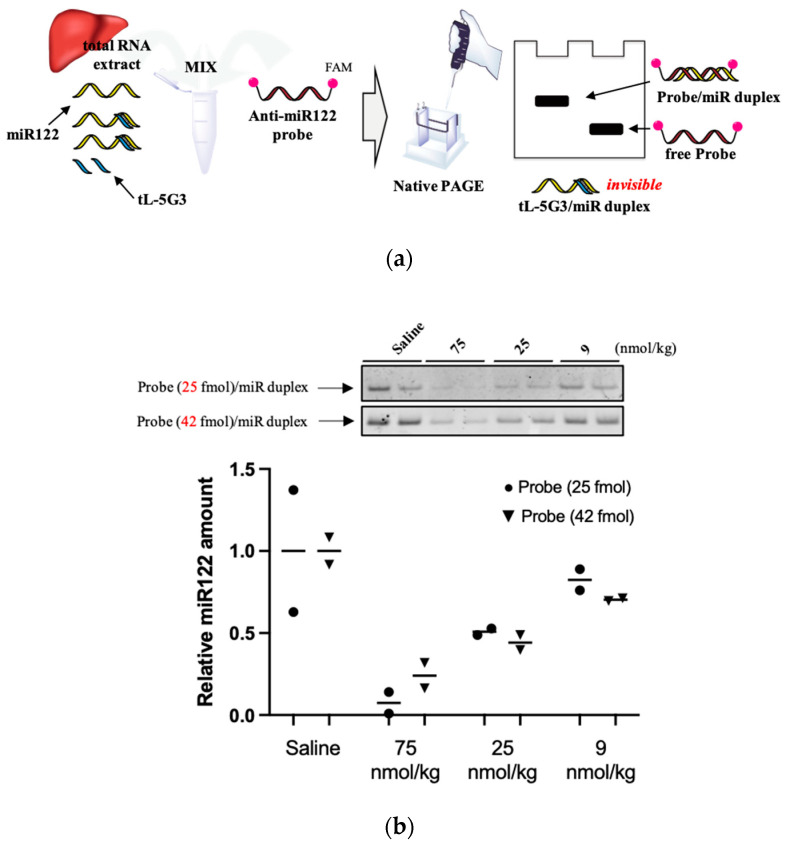
(**a**) Schematic illustration of the miRNA-122 detection method devised in this study. (**b**) Representative gel images of the miR122 detection system devised in this study. Total RNA samples obtained from murine liver fragments were analyzed using this detection technique. Total RNA (25 µg) and the FAM-conjugated anti-miR122 probe (25 or 42 fmol) were used in this study. Bands are visualized using Image Quant LAS4000 (GE Healthcare, IL). The fluorescence intensity of the bands was quantified using ImageJ software. The relative amount was estimated from the relative intensity of each duplex band, normalized with the averaged intensity of the duplex bands obtained from each saline-treated arm.

Due to their small size and high sequence similarity within families, the detection of miRNA expression is not as straightforward as that of other long RNAs [32]. To further confirm the sequestering of miR-122 by **tL-5G3**, we devised a Northern blotting-like detection method to visualize miRNA-122. Briefly, as shown in Figure 3a, total RNA extracted from the liver samples in each treatment arm was co-incubated with a fully-miRNA-122 complementary DNA/LNA mixmer probe (**Anti-miR122-probe**, Table 1) with a FAM fluorophore at both ends. The probe is supposed to bind tightly to free miRNA-122 and not to **tL-5G3**-bound miRNA-122. We then performed native polyacrylamide electrophoresis to detect the duplex band containing miRNA-122 and the probe. After optimizing the probe concentration, we selected two amounts of **anti-miR122 probe** (25 and 42 fmol) and successfully visualized the duplex bands very clearly (Figure 3b). The dose-responsive reduction of the duplex bands was observed on both gels, and the trend was found to be in accordance with the results obtained from the SplintR^®^ qPCR analysis (Figure 2c), although a slight fluctuation in the band intensity was observed with this method. This was probably due to the fluctuation in the annealing conditions and/or the molar equivalence of the probe against miRNA-122, because the probe used in this method can bind to miR122 more strongly than those used in SplintR^®^ PCR. However, this result supports the antisense inhibitory mechanism of **tL-5G3**.

### 3.4. Configuration–Activity Study of GalNAc-Conjugated Tiny LNA

To gain more insight into the configuration (or structure)–activity relationship of the GalNAc-conjugated tiny LNAs, we designed and synthesized several other constructs to test their activity in vivo. As listed in Table 1, **tL-5G1** is similar to **tL-5G3**, but has only one GalNAc moiety at the 5′ terminus. This was designed based on our previous findings on gapmer-type ASOs, in which the introduction of a single GalNAc molecule can improve the in vivo activity of ASOs [18]. **tL-5G3-PO** is a fully phosphodiester-linked, tiny LNA. This was designed to explore the possibility of lifting the phosphorothioate requirement, which was frequently alleged to limit the possibility of ASO therapy, by taking advantage of the tiny LNA fully armed by nuclease resistant 2′,4′-BNA/LNA modification. In contrast, **tL-5G3-PS** is a fully phosphorothioate-modified version of **tL-5G3**. Our previous study showed that utilizing phosphorothioate linkages as GalNAc conjugation linkers generally abrogate or weaken ligand activity. **tL-5G3-PS** was prepared to test whether a similar trend could be observed in this different class of ASOs. Lastly, the GalNAc*_hp_* units were introduced into the 3′-end of the tiny LNA, called **tL-3G3**. According to some studies, 5′-GalNAc-conjugated ASOs are likely to be more potent than their 3′-conjugated counterparts [18,33].

**Figure 4:**
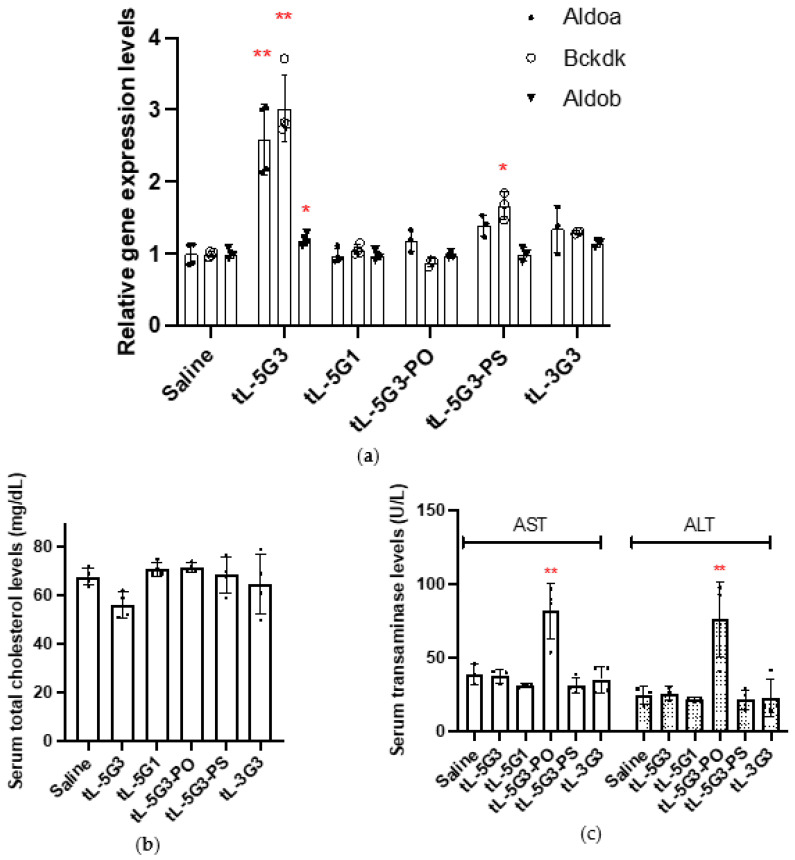
In vivo structure–activity relationship study. (**a**) The expression levels of miRNA-122-targeted or non-targeted mRNAs in the liver of mice (6-week-old male C57Bl6/J, *n* = 4), 7 days after a single injection of each construct at a dosage of 12.5 nmol/kg. (**b**,**c**) Their corresponding serum cholesterol levels and liver transaminase levels, respectively. ** *p* < 0.01 and * *p* < 0.05 (vs. saline).

Each mouse (C57BL/6J, male, *n* = 4) was subjected to a single subcutaneous injection of each drug at a dose of 12.5 nmol/kg, a little more than the ED50 dose for **tL-5G3**. Seven days after injection, the mice were sacrificed to analyze their mRNA expression profiles and serum cholesterol and liver transaminase levels. In addition to **tL-5G3**, **tL-5G3-PS** exhibited a statistically significant increase in Bckdk mRNA levels (* *p* = 0.0112). Although not statistically significant, **tL-5G3-PS** and **tL-3G3** showed a slight increase in Aldoa mRNA, while the expression of the two miRNA-122 target genes was unchanged by **tL-5G1** and **tL-5G3-PO** at this dosage, which was backed by the change in serum total cholesterol levels (Figure 4a,b). These results suggest that conjugation to the 5′ terminus is more favorable for eliciting the ligand activity when comparing conjugation at the 5′ and 3′ ends. This result was consistent with a few previous observations of GalNAc-conjugated RNase H-mediated ASOs [18,33]. According to our research reported elsewhere [21], we speculate that this difference in activity between the 5′- and 3′-congeners originated from their differences in sensitivity (tolerability) towards nucleases. The predominant nuclease activity in the serum is known to be 3′-exonuclease, while the predominant activity in cells is endonuclease [29,34]. Therefore, the 5′-congener could be more stable in the circulation than its 3′ counterpart. In terms of the number of ligands incorporated, the more ligands incorporated, the better the distribution of the tiny LNA to the liver [18]. One possibility is that a tiny LNA would require a greater number of GalNAc units than gapmer ASOs because of its poorer pharmacokinetics for the above-mentioned reasons (Section 3.2). Moreover, it is quite intriguing to see that, using a biolabile phosphodiester bond on the tiny LNA is more advantageous than a metabolically stable phosphorothioate linkage (e.g., **tL-5G3** vs. **tL-5G3-PS**). This implies the involvement of endogenous endonucleases to digest the PO linkage of the conjugate [21]. If this is the case, even a tiny LNA, a very small ASO fully armed with nuclease resistant 2′,4′-BNA/LNA, is meant to be a substrate of the responsible endonuclease (s). This notion is further supported by the increase in transaminase levels associated with **tL-5G3-PO** (Figure 4c). This trend in **tL-5G3-PO** hepatotoxicity was suspected to be derived from the metabolites of **tL-5G3-PO**, meaning that tiny LNA could be further digested in pieces after being taken up by the liver. This onset of hepatotoxicity, possibly from the shorter fragments of the putative tiny LNAs, could also be interesting, since the toxicity of 2′,4′-BNA/LNA-based ASOs has long been alleged, yet remains unsolved [35,36,37].

### 3.5. Biodistribution Study of GalNAc-Conjugated Tiny LNA

**Figure 5:**
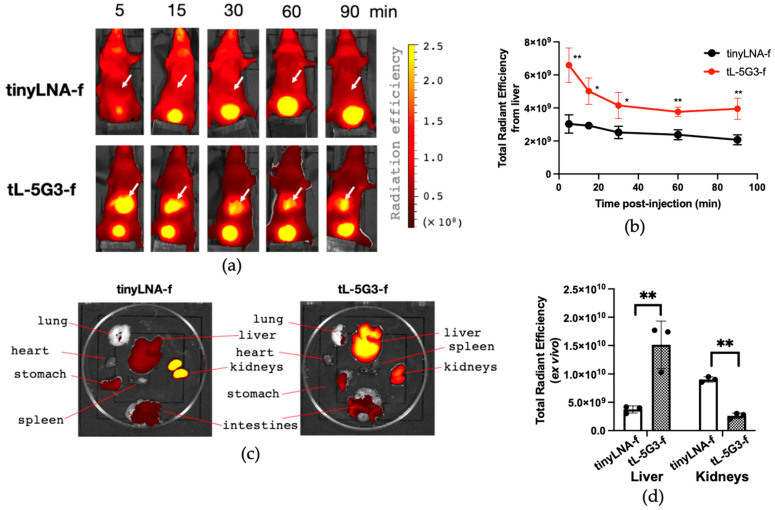
In vivo and ex vivo visualization of the effect of GalNAc-conjugation on biodistribution of tiny LNA. (**a**) Representative fluorescence images of mice (Balb/cSlc-nu/nu, male, 6-week-old, *n* = 3) at different time points after administration of each Alexa647-labeled oligonucleotide (300 pmol) via tail vein (See Supplementary Figure S4 for all images). Arrows point to the liver. (**b**) Semi-quantitative fluorescence analysis of the liver of each mouse using IVIS imager (Total Radiation efficiency = (photons/sec)/(µW/cm^2^)). (**c**) Ex vivo images of representative tissues from mice after 90 min of administration (See Supplementary Figure S5 for all images). (**d**) Semi-quantitative fluorescent analysis for the tissue accumulation of each tiny LNA in the liver and kidneys using IVIS. Each fluorescence image was overlayed with a corresponding photograph. For (**b**,**d**), a multiple unpaired *t*-test was utilized. ** *p* < 0.01 and * *p* < 0.05.

To further support the pharmacokinetic benefit of GalNAc conjugation on tiny LNAs, we pursued an in vivo fluorescent imaging study with GalNAc-conjugated and -unconjugated fluorescently labeled oligonucleotides, **tL-5G3-f** and **tinyLNA-f** (Table 1). For in vivo imaging application, Alexa Fluor^®^ 647, a tissue penetrant far-red fluorescent dye, was introduced into the corresponding amine linker-bearing oligonucleotide on its 3′ terminal, in which a phosphate linkage between the ASOs and the amino linker has a biostable phosphorothioate modification to prohibit the labeling dye from digestion in the body during the imaging experiment. Then, the conjugation of the fluorophore to each oligonucleotide was conducted post-synthetically in a buffered condition using *N*-hydroxysuccinimide-activated Alexa Fluor^®^ 647. The resulting tiny LNAs were purified by de-salting columns followed by reverse-phase HPLC (Supplementary Figures S2 and S3). Identification was carried out on MALDI-TOF-MS. Mice (Balb/cSlc-nu/nu, male, 6-week-old, *n* = 3) were then subjected to a single intravenous injection of either **tinyLNA-f** or **tL-5G3-f** (300 pmol/mouse), and the time-lapse image capturing of the whole body was initiated 5 min after the intravenous injection and lasted for 90 min. A representative image at each time point for both treatment groups is exhibited in Figure 5a. The unconjugated **tinyLNA-f** was found delocalized and seen as vague fluorescence throughout the body. A strong signal was only observed in the bladder during the course of the experiment. On the other hand, along with the bladder, the liver strongly glowed since right after the injection of **tL-5G3-f** as we expected (Figure 5a). Semi-quantitative fluorescence analysis showed that **tL-5G3-f** was statistically significant compared to **tinyLNA-f** for the amount accumulated in the liver at each time point. The radiation efficiency of **tL-5G3-f** from the liver gradually decreased even during the 90 min of observation (Figure 5b). Since a consistent and significantly stronger signal of **tL-5G3-f** from the liver was still observed at the 90 min time point of administration, we decided to sacrifice the mice and perform ex vivo imaging to further confirm that the origin of the fluorescence was the liver. As shown in Figure 5c, while the strongest radiation of **tinyLNA-f** was observed in the kidneys, the highest total radiant efficiency was found in the liver for **tL-5G3-f**. Semi-quantitative analysis showed that GalNAc conjugation increased the accumulation in the liver by about 5-fold, while it decreased the accumulation in the kidney by about one-fifth. The latter may also be beneficial in terms of reducing nephrotoxicity. This change in distribution pattern between liver and kidney is in good agreement with our previous observations on GalNAc-conjugated 13mer LNA gapmer [22]. The actual fold changes that we obtained in this study, although they are not quite accurate, are almost consistent with those obtained from the previous study. However, as seen in Figure 5a,c, most of **tinyLNA-f** localizes in the kidneys or the bladder and the liver fluorescence seems almost a background (or autofluorescence) level. This could mean that tiny LNAs can be more easily eliminated from the circulation than the ones with a longer base sequence. We found that GalNAc conjugation has clearly improved the poor pharmacokinetics of tiny LNAs by avoiding their distribution to the kidneys. Further precise and quantitative pharmacokinetic analysis is required to fully explain this large benefit of GalNAc conjugation on tiny LNAs observed in this study.

## 4. Conclusions

In this study, we first demonstrated the effect of GalNAc conjugation on anti-miRNA ASOs, specifically on tiny LNAs. The impact of the conjugation on their in vivo potency was estimated to be ~300–500 folds, which was much larger than we expected from previous studies of GalNAc-conjugated gapmer-type ASOs. We have also confirmed in this study that this large benefit of GalNAc conjugation on tiny LNAs can be ascribed to the improvement of their naturally poor pharmacokinetic properties. To make the most out of GalNAc conjugation, the ligand should be introduced into its 5′ end rather than the 3′ end via a biolabile phosphodiester bond. This result suggests that tiny LNAs could be recognized as endonucleases and required digestion to liberate the parent tiny LNA at an appropriate time. We believe that when full metabolic profiles are obtained and the optimal ligands are already determined, ligand-conjugated anti-miRNA ASOs would pave the way for a clinically useful anti-miRNA therapy.

## Figures and Tables

**Table 1:** Oligonucleotides used in this study.

ID	Sequence (5′ to 3′)
**CtrASO** ^(a)^ (apoB-targeting cholesterol-lowering positive control)	HºHºHºG^C^a^t^t^g^g^t^a^t^T^C^A
**tinyLNA**	C^A^C^A^C^T^C^C
**tL-5G3**	HºHºHºC^A^C^A^C^T^C^C
**tinyCtr-5G3** ^(b)^	HºHºHºT^C^A^T^A^C^T^A
**tL-5G1**	HºC^A^C^A^C^T^C^C
**tL-5G3-PO**	HºHºHºCºAºCºAºCºTºCºC
**tL-5G3-PS**	H^H^H^C^A^C^A^C^T^C^C
**tL-3G3**	C^A^C^A^C^T^C^CºHºHºH
**Anti-miR122-probe**	5′-FºaºcºAºaºaºCºaºcºCºaºtºTºgºtº CºaºcºAºcºtºCºcºaºF-3′
**tinyLNA-f** ^(b)^	C^A^C^A^C^T^C^C^f
**tL-5G3-f** ^(b)^	HºHºHºC^A^C^A^C^T^C^C^f

Upper and lower case letters indicate 2′,4′-BNA/LNA and native DNA, respectively. H represents monovalent GalNAc. ^ and º indicate a phosphorothioate (PS) linkage and phosphodiester (PO) linkage, respectively. F and f denote the FAM and Alexa Fluor^TM^ 647 fluorophores, respectively. ^(a)^ A GalNAc-conjugated apolipoprotein-B targeting ASO that we have previously reported as a highly potent ASO [18], which was used here as a potent cholesterol-lowering positive control. All oligonucleotides were synthesized by Gene Design Inc., except for those labeled as ^(b)^.

## Supplementary Materials

The following are available online at https://www.mdpi.com/article/10.3390/pharmaceutics13060817/s1. Figure S1: HPLC analysis of **tinyCtr-5G3**. Figure S2: HPLC analysis of **tinyLNA-f**. Figure S3: HPLC analysis of **tL-5G3-f**. Figure S4: In vivo fluorescent images for all mice tested. Figure S5: Ex vivo fluorescent images for all mice tested.

## References

[1] Treiber T., Treiber N., Meister G. (2019). Regulation of microRNA biogenesis and its crosstalk with other cellular pathways. Nat. Rev. Mol. Cell Biol..

[2] Baumann V., Winkler J. (2014). miRNA-based therapies: Strategies and delivery platforms for oligonucleotide and non-oligonucleotide agents. Future Med. Chem..

[3] McDermott A.M., Heneghan H.M., Miller N., Kerin M.J. (2011). The Therapeutic Potential of MicroRNAs: Disease Modulators and Drug Targets. Pharm. Res..

[4] Esau C., Davis S., Murray S.F., Yu X.X., Pandey S.K., Pear M., Watts L., Booten S.L., Graham M., McKay R. (2006). miR-122 regulation of lipid metabolism revealed by in vivo antisense targeting. Cell Metab..

[5] Krützfeldt J., Rajewsky N., Braich R., Rajeev K.G., Tuschl T., Manoharan M., Stoffel M. (2005). Silencing of microRNAs in vivo with ‘antagomirs’. Nat. Cell Biol..

[6] Jopling C.L., Schütz S., Sarnow P. (2008). Position-Dependent Function for a Tandem MicroRNA miR-122-Binding Site Located in the Hepatitis C Virus RNA Genome. Cell Host Microbe.

[7] Henke J.I., Goergen D., Zheng J., Song Y., Schüttler C.G., Fehr C., Jünemann C., Niepmann M. (2008). microRNA-122 stimulates translation of hepatitis C virus RNA. EMBO J..

[8] Fukuhara T., Matsuura Y. (2012). Role of miR-122 and lipid metabolism in HCV infection. J. Gastroenterol..

[9] Jopling C.L., Yi M., Lancaster A.M., Lemon S.M., Sarnow P. (2005). Modulation of Hepatitis C Virus RNA Abundance by a Liver-Specific MicroRNA. Science.

[10] Bajan S., Hutvagner G. (2020). RNA-Based Therapeutics: From Antisense Oligonucleotides to miRNAs. Cells.

[11] Elmén J., Lindow M., Schütz S., Lawrence M., Petri A., Obad S., Lindholm M.W., Hedtjärn M., Hansen H.F., Berger U. (2008). LNA-mediated microRNA silencing in non-human primates. Nat. Cell Biol..

[12] Kojima S., Gatfield D., Esau C.C., Green C.B. (2010). MicroRNA-122 Modulates the Rhythmic Expression Profile of the Circadian Deadenylase Nocturnin in Mouse Liver. PLoS ONE.

[13] Stelma F., Van Der Ree M.H.M.H., Sinnige M.J., Brown A., Swadling L., De Vree J.M.L., Willemse S.B., Van Der Valk M., Grint P., Neben S. (2017). Immune phenotype and function of natural killer and T cells in chronic hepatitis C patients who received a single dose of anti-MicroRNA-122, RG-101. Hepatology.

[14] Yoshioka K., Kunieda T., Asami Y., Guo H., Miyata H., Yoshida-Tanaka K., Sujino Y., Piao W., Kuwahara H., Nishina K. (2019). Highly efficient silencing of microRNA by heteroduplex oligonucleotides. Nucleic Acids Res..

[15] Obad S., Dos Santos C.O., Petri A., Heidenblad M., Broom O., Ruse C., Fu C., Lindow M., Stenvang J., Straarup E.M. (2011). Silencing of microRNA families by seed-targeting tiny LNAs. Nat. Genet..

[16] Rottiers V., Obad S., Petri A., McGarrah R., Lindholm M.W., Black J.C., Sinha S., Goody R.J., Lawrence M.S., Delemos A.S. (2013). Pharmacological Inhibition of a MicroRNA Family in Nonhuman Primates by a Seed-Targeting 8-Mer AntimiR. Sci. Transl. Med..

[17] Debacker A.J., Voutila J., Catley M., Blakey D., Habib N. (2020). Delivery of Oligonucleotides to the Liver with GalNAc: From Research to Registered Therapeutic Drug. Mol. Ther..

[18] Yamamoto T., Sawamura M., Wada F., Harada-Shiba M., Obika S. (2016). Serial incorporation of a monovalent GalNAc phosphoramidite unit into hepatocyte-targeting antisense oligonucleotides. Bioorganic Med. Chem..

[19] Rajeev K.G., Nair J.K., Jayaraman M., Charisse K., Taneja N., O’Shea J., Willoughby J.L.S., Yucius K., Nguyen T., Shulga-Morskaya S. (2015). Hepatocyte-Specific Delivery of siRNAs Conjugated to Novel Non-nucleosidic TrivalentN-Acetylgalactosamine Elicits Robust Gene Silencing in Vivo. ChemBioChem.

[20] Matsuda S., Keiser K., Nair J.K., Charisse K., Manoharan R.M., Kretschmer P., Peng C.G., Kel’In A.V., Kandasamy P., Willoughby J.L. (2015). siRNA Conjugates Carrying Sequentially Assembled Trivalent N-Acetylgalactosamine Linked Through Nucleosides Elicit Robust Gene Silencing In Vivo in Hepatocytes. ACS Chem. Biol..

[21] Yamamoto T., Sawamura M., Terada C., Kashiwada K., Wada F., Yamayoshi A., Obika S., Harada-Shiba M. (2020). Effect of modular conjugation strategy forN-acetylgalactosamine-targeted antisense oligonucleotides. Nucleosides Nucleotides Nucleic Acids.

[22] Wada F., Yamamoto T., Ueda T., Sawamura M., Wada S., Harada-Shiba M., Obika S. (2018). Cholesterol–GalNAc Dual Conjugation Strategy for Reducing Renal Distribution of Antisense Oligonucleotides. Nucleic Acid Ther..

[23] Yamamoto T., Terada C., Kashiwada K., Yamayoshi A., Harada-Shiba M., Obika S. (2019). Synthesis of MonovalentN-Acetylgalactosamine Phosphoramidite for Liver-Targeting Oligonucleotides. Curr. Protoc. Nucleic Acid Chem..

[24] Jin J., Vaud S., Zhelkovsky A.M., Posfai J., McReynolds L.A. (2016). Sensitive and specific miRNA detection method using SplintR Ligase. Nucleic Acids Res..

[25] Elmén J., Lindow M., Silahtaroglu A., Bak M., Christensen M., Lind-Thomsen A., Hedtjärn M., Hansen J.B., Hansen H.F., Straarup E.M. (2007). Antagonism of microRNA-122 in mice by systemically administered LNA-antimiR leads to up-regulation of a large set of predicted target mRNAs in the liver. Nucleic Acids Res..

[26] Sewing S., Gubler M., Gérard R., Avignon B., Mueller Y., Braendli-Baiocco A., Odin M., Moisan A. (2019). GalNAc Conjugation Attenuates the Cytotoxicity of Antisense Oligonucleotide Drugs in Renal Tubular Cells. Mol. Ther. Nucleic Acids.

[27] Janas M.M., Schlegel M.K., Harbison C.E., Yilmaz V.O., Jiang Y., Parmar R., Zlatev I., Castoreno A., Xu H., Shulga-Morskaya S. (2018). Selection of GalNAc-conjugated siRNAs with limited off-target-driven rat hepatotoxicity. Nat. Commun..

[28] Prakash T.P., Graham M.J., Yu J., Carty R., Low A., Chappell A., Schmidt K., Zhao C., Aghajan M., Murray H.F. (2014). Targeted delivery of antisense oligonucleotides to hepatocytes using triantennary N-acetyl galactosamine improves potency 10-fold in mice. Nucleic Acids Res..

[29] Post N., Yu R., Greenlee S., Gaus H., Hurh E., Matson J., Wang Y. (2019). Metabolism and Disposition of Volanesorsen, a 2′-O-(2 methoxyethyl) Antisense Oligonucleotide, Across Species. Drug Metab. Dispos..

[30] Watanabe T.A., Geary R.S., Levin A.A. (2006). Plasma Protein Binding of an Antisense Oligonucleotide Targeting Human ICAM-1 (ISIS 2302). Oligonucleotides.

[31] Kilanowska A., Studzińska S. (2020). In vivoandin vitrostudies of antisense oligonucleotides—A review. RSC Adv..

[32] Huang Y., Zou Q., Wang S.P., Tang S.M., Zhang G.Z., Shen X.J. (2011). The discovery approaches and detection methods of microRNAs. Mol. Biol. Rep..

[33] Østergaard M.E., Yu J., Kinberger G.A., Wan W.B., Migawa M.T., Vasquez G., Schmidt K., Gaus H.J., Murray H.M., Low A. (2015). Efficient Synthesis and Biological Evaluation of 5′-GalNAc Conjugated Antisense Oligonucleotides. Bioconjugate Chem..

[34] Shaw J.-P., Kent K., Bird J., Fishback J., Froehler B. (1991). Modified deoxyoligonucleotides stable to exonuclease degradation in serum. Nucleic Acids Res..

[35] Burel S.A., Hart C.E., Cauntay P., Hsiao J., Machemer T., Katz M., Watt A.T., Bui H.-H., Younis H., Sabripour M. (2016). Hepatotoxicity of high affinity gapmer antisense oligonucleotides is mediated by RNase H1 dependent promiscuous reduction of very long pre-mRNA transcripts. Nucleic Acids Res..

[36] Swayze E.E., Siwkowski A.M., Wancewicz E.V., Migawa M.T., Wyrzykiewicz T.K., Hung G., Monia B.P., Bennett C.F. (2006). Antisense oligonucleotides containing locked nucleic acid improve potency but cause significant hepatotoxicity in animals. Nucleic Acids Res..

[37] Hagedorn P.H., Yakimov V., Ottosen S., Kammler S., Nielsen N.F., Høg A.M., Hedtjärn M., Meldgaard M., Møller M.R., Ørum H. (2013). Hepatotoxic Potential of Therapeutic Oligonucleotides Can Be Predicted from Their Sequence and Modification Pattern. Nucleic Acid Ther..

