# Supplementary material for: Highly Potent GalNAc-Conjugated Tiny LNA Anti-miRNA-122 Antisense Oligonucleotides
Source: Pharmaceutics. 2021 May 31;13(6):817. doi: 10.3390/pharmaceutics13060817 (PMC8228246; doi:10.3390/pharmaceutics13060817)
Supplement: Supplementary file 1 [file pharmaceutics-13-00817-s001.zip › pharmaceutics-1191355-supplementary.pdf]

pharmaceutics

MDPI

Supplementary Materials: Highly Potent GalNAc-Conjugated tiny LNA Anti-miRNA-122 Antisense Oligonucleotides

Tsuyoshi Yamamoto, Yahiro Mukai, Fumito Wada, Chisato Terada, Yukina Kayaba, Kaho Oh, Asako Yamayoshi, Satoshi Obika and Mariko Harada–Shiba

- **Figure S1** HPLC analysis of **tinyCtr-5G3**
- **Figure S2** HPLC analysis of **tinyLNA-f**
- **Figure S3** HPLC analysis of **tL-5G3-f**
- **Figure S4** In vivo fluorescent images for all mice tested.
- **Figure S5** Ex vivo fluorescent images for all mice tested.

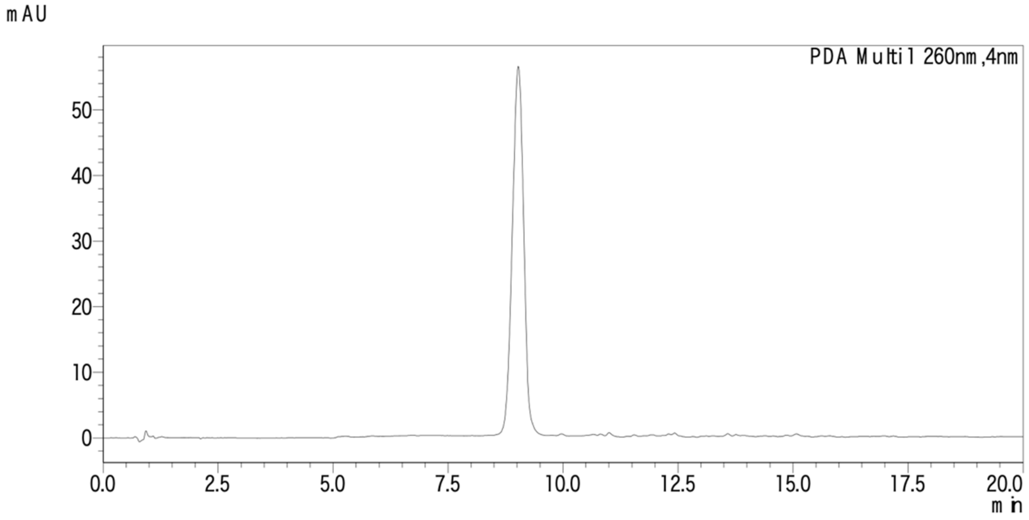

Figure S1. HPLC analysis of tinyCtr-5G3.

.

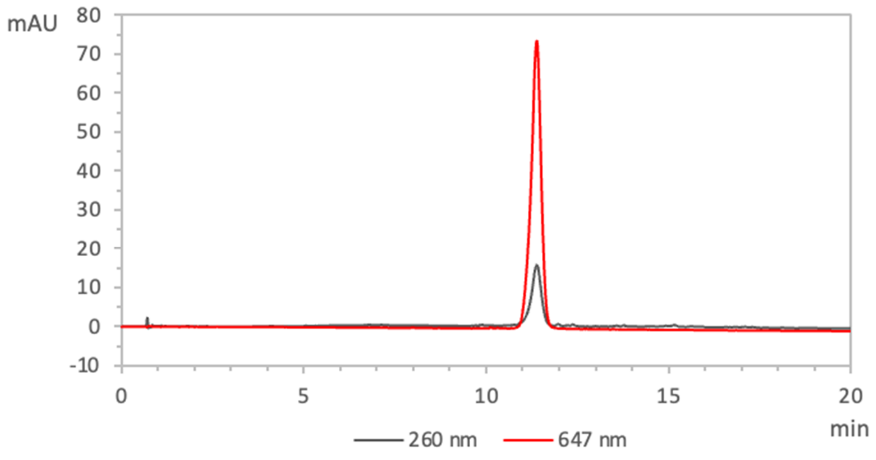

Figure S2. HPLC analysis of tinyLNA-f.

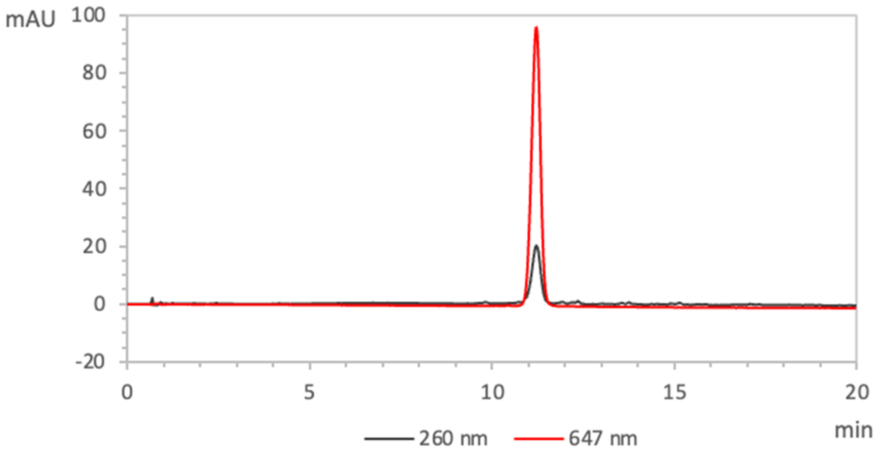

Figure S3. HPLC analysis of tL-5G3-f.

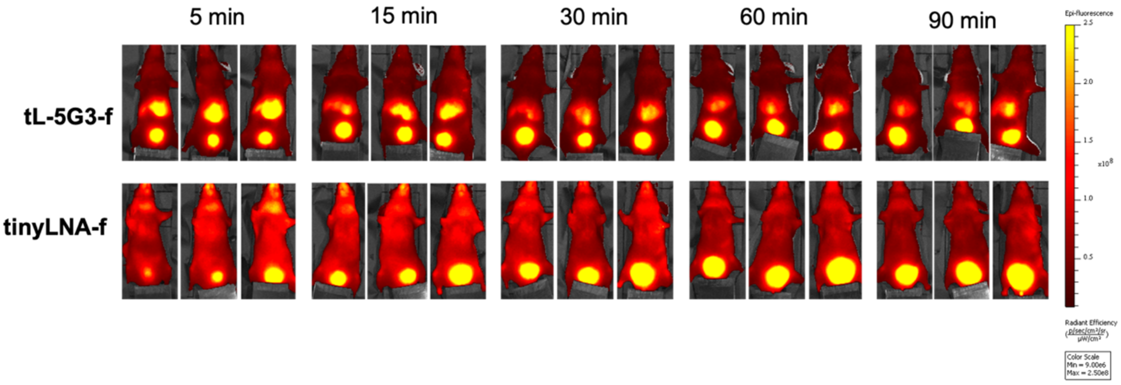

Figure S4. In vivo fluorescent images for all mice tested.

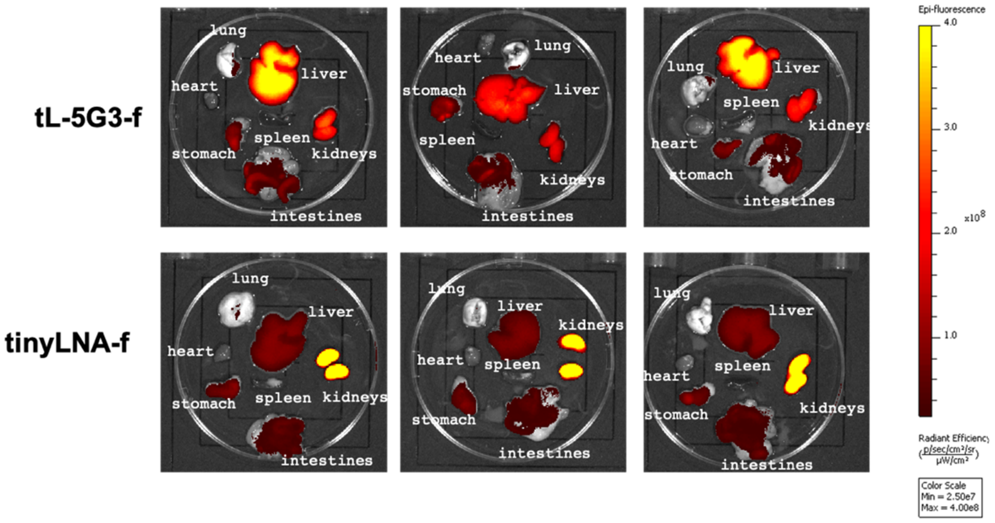

Figure S5. Ex vivo fluorescent images for all mice tested.
